# Light curing infection control barriers: do some types jeopardize the concept of conventional bulk-fill composites?

**DOI:** 10.1186/s12903-024-05033-8

**Published:** 2024-11-03

**Authors:** Dalia I. Sherief, Mohamed M. Kandil, Dina Ahmed El-Refai

**Affiliations:** 1 Biomaterials Department, Faculty of Dentistry, Ain-Shams University, Organization of African unity, Cairo, Egypt; 2 Badya University, Badya, Giza, Egypt; 3 Biomaterials Department, Faculty of Dentistry, Galala University, Suez, Egypt

**Keywords:** Infection control barriers, Bulk- fill composites, Flexural strength, Light irradiance, Degree of conversion, Water vapor permeability

## Abstract

**Background:**

Using infection control barriers (ICBs) on light curing units (LCUs) became mandatory to achieve proper infection control measures without jeopardizing the integrity of the restorations, especially at deeper layers. This study explored the effect of two ICBs on the irradiance of the LCU, as well as the degree of conversion (DC) and flexural strength (FS) of two types of bulk-fill composites. Water vapor permeability (WVP) of both barriers was also assessed to evaluate the capability of such barriers to prevent transmission of blood and saliva droplets and aerosols.

**Methods:**

Two bulk-fill composites (X-tra fil and Tetric N- ceram) and two ICBs (Pinnacle Cure sleeve and Sanita wrapping film) were used in this study. Light irradiance was recorded per experimental condition using spectroradiometer. For DC and FS, specimens of 4 mm thickness were prepared. Each specimen was composed of two separable upper and lower layers of thickness 2 mm. DC and FS were measured using Infra-red spectroscopy and three-point loading test respectively. WVP was investigated using the cup method. Means and standard deviations were calculated, and the data were statistically analyzed using factorial analysis of variance test (α = 0.05).

**Results:**

Light irradiance showed highest values using no ICBs and lowest values using Pinnacle curing sleeve. Both bulk-fill composites showed higher DC mean values without ICBs and when using Sanita wrapping film for both upper and lower layers of the specimens compared to Pinnacle curing sleeve. The upper layers of composite specimens showed higher DC compared to lower layers for all experimental conditions. Both ICBs had no adverse effect on FS of both composites’ upper layers. Pinnacle sleeve significantly reduced FS of both composites’ lower layers. X-tra fil showed higher DC and FS compared to Tetric N-Ceram for all experimental conditions. Regarding WVP; the wrapping film showed higher WVP compared to the curing sleeve.

**Conclusions:**

Sanita wrapping film can be used as a successful ICB, without jeopardizing the concept of bulk-fill composites. Pinnacle cure sleeve can be considered an effective ICB, however its influence on properties and serviceability of bulk-fill composites remains questionable.

## Background

Dental professionals are subjected to a great diversity of microorganisms existing in the saliva and blood of patients. Avoiding cross-infection in the dental office is necessary for the patient’s dental care. Practicing effective infection control measures inhibits cross-contamination among patients and the dental health care team [1].

Along with the Centers for Diseases Control and Prevention strategies, the light curing unit (LCU) is considered a semi-critical instrument due to its indirect contact with mucous membranes posing the likelihood of infection transmission.

The body, light guide, and the control buttons on the LCU can be a source of contamination once they become infected when used on a patient [2]. Using protective infection control barriers (ICBs) on LCUs has become mandatory to prevent direct contact between the oral tissues and the light guide, hence avoiding transmitting diseases such as Hepatitis B, Acquired Immune Deficiency Syndrome, and, more recently, COVID-19 [3, 4]. They also eliminate the need for autoclaving or chemical disinfection of the light guide that may cause its damage [5].

ICBs also protect the light tip from being contaminated with uncured resin. It has been stated that the light tips of 35–68% of the LCUs in dental offices have bonding agent or resin adhering to them when used without ICBs [6]. Such resin debris will decrease the light output and consequently have an adverse effect on the resin polymerization [7].

Numerous translucent ICBs have been used with LCUs as plastic wrapping films and custom fitting curing sleeves. Barriers are constructed from different materials, including low-density polyethylene, latex-free polyurethane, and polyvinyl chloride [8].

Questions have been raised concerning the use of such ICBs on the light-curing units tips and the likelihood of interference with output light power. Reduction in power density of light sources impedes effective polymerization of resins leading to the presence of a non-polymerized or partially polymerized composite resin restorative material. Inadequate polymerization of the composite resin is blamable for the water sorption increase, staining, decrease of surface hardness, recurrent caries thus resulting in failure of the restoration [9, 10].

Several of the above-mentioned barriers were investigated for their effect on light intensity of LCUs. Since the degree of polymerization of composite restorations is already reduced by light attenuation while traversing the resin composite material [6, 11], any additional decrease of light intensity caused by ICBs has the capability to further decrease composites’ curing depth [8].

Most research urged the use of properly positioned smooth ICBs over the end of the light guide since it caused minimal decrease in power density of light sources. Such decrease had no adverse effect on the clinical performance of the restoration [8, 12, 13]. However, to the best of our knowledge no studies evaluated the effect of these barriers on resin composite increments more than 2 mm thick, as in the case of bulk-fill composites which are designed to be placed in 4- to 5-mm increments to save significant time while restoring large and deep cavities.

Consequently, the aim of this study was to evaluate the effect of two ICBs covering the LCU tip, on the irradiance (mW/cm^2^) of a light curing unit as well as the degree of conversion and flexural strength of two bulk-fill composites (X-tra fil and tetric N-ceram). Water vapor permeability of both barriers was also explored to assess their efficacy as ICBs.

The null hypotheses were that the two infection control barriers tested (1) have no influence on irradiance of the curing unit (2) have no effect on the degree of conversion and flexural strength of the two bulk-fill composites (3) have the same water vapor permeability effect.

## Materials and methods

The present research was conducted in the Biomaterials Department, Faculty of Dentistry. While this was an in vitro study, which did not involve any dealings with human contributors or animals, ethical exemption was provided by the university ethical committee (FDASU-Rec ER092306).

Two disposable infection control barriers were investigated: A commercially available barrier (Pinnacle Cure Sleeve, Kerr, USA) made of low-density polyethylene and a multi-purpose wrapping film (Sanita Consumer products) made of polyvinyl chloride (PVC). Two bulk-fill composites of universal shade (X-tra fil, Voco, Germany) (Tetric N-ceram, Ivoclar Vivadent) were used in this study.

The light curing unit used was Bluephase N (Ivoclar Vivadent); a polywave LED light curing unit with spectrum of 385 to 515 nm and an intensity of 1200mW/cm^2^.The light guide was of 10 mm diameter.

### Light irradiance

The irradiance (mW/cm^2^) of the light curing unit was measured using a bench-top spectroradiometer (C Series flexOptometers, Gamma Scientific, USA). According to the manufacturer, the C Series flexOptometer is designed for measurement of irradiance less than 0.055 nanoWatts/cm² to 8000 microWatts/cm² and wavelength range of 200–2600 nm. To ascertain the light intensity produced by the light-cure unit, the light output was assessed after ten seconds to grant the steadiness of the curing unit after activation. Five measurements were taken using each of the disposable barrier options (cure sleeve, wrapping film) and without using ICBs. Care was taken not to place the seam of Pinnacle cure sleeve over the light tip. As for the Sanita wrapping film it was correctly placed on the light tip without any folds or creases. The spectral irradiance delivered using both barriers was also detected.

### Degree of conversion of bulk- fill composite

**Fig. 1:**
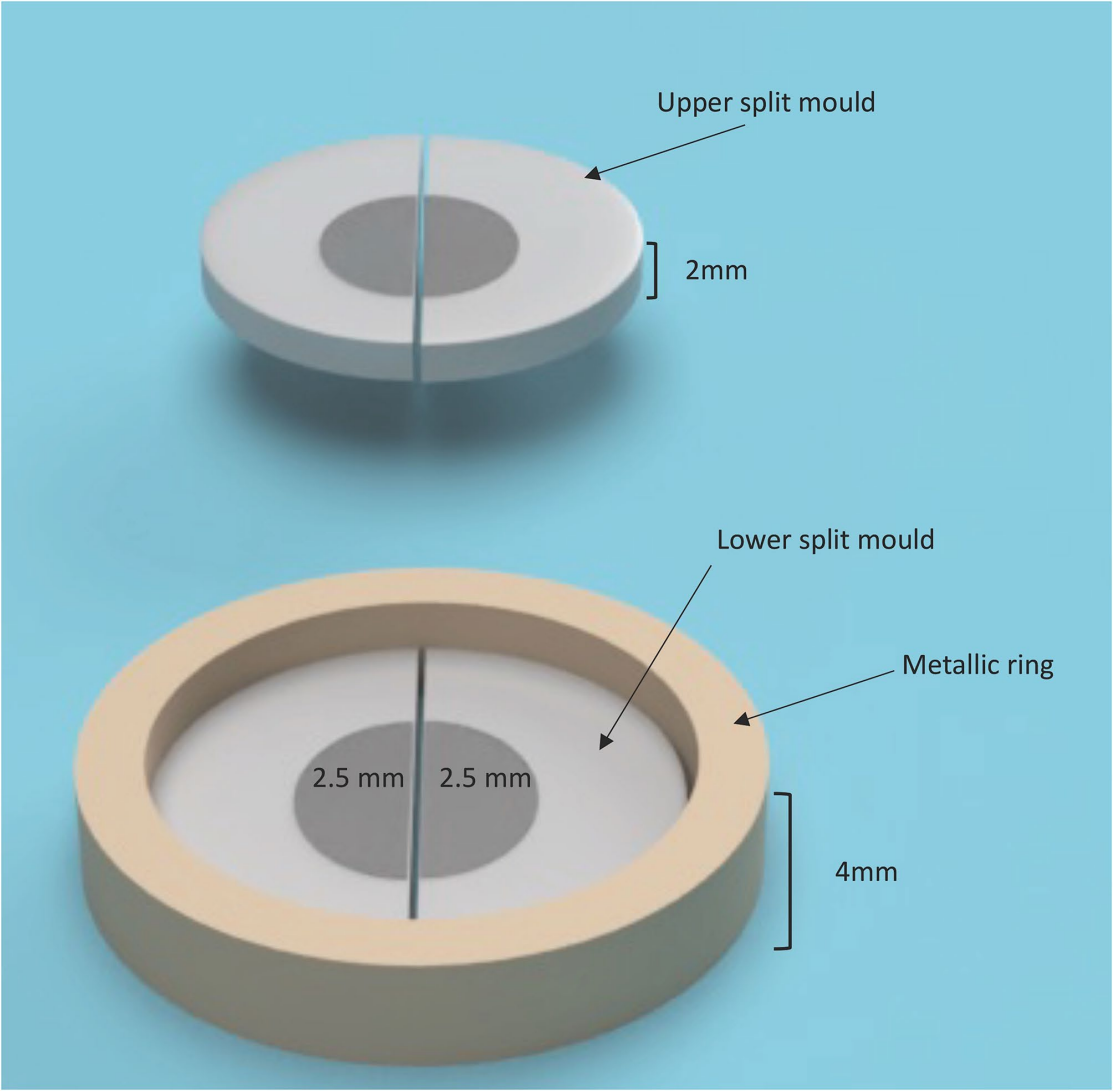
Schematic representation of molds assembly used in the construction of degree of conversion specimens

Composite discs were prepared for each type of bulk-fill composite using an assembly of two split circular Teflon molds (5 mm diameter,2 mm thickness) stacked on top of each other to mimic a 4-mm-deep cavity (Fig. 1). The composite was packed in the first mold and covered with a polyester strip (Mylar, 0.05 mm thick; DuPont, Wilmington, DE). The second mold was then placed on top and secured in place using a 4 mm metallic ring then filled with composite and covered with another polyester strip.

The assembly was photoactivated from above for 20 s. The presence of the polyester strips allowed separating the molds after curing to produce 2 composite discs available for degree of conversion (DC) testing. Each specimen was labelled to distinguish upper from lower discs. This procedure was performed six times for each bulk-fill composite using the LCU with and without ICBs. Six 4 mm thick specimens were obtained for each experimental condition (2 mm upper disc and 2 mm lower disc for each specimen). The total number of specimens was 36 while the total number of 2 mm thick discs was 72. Degree of conversion (DC) for each disc layer was assessed via transmission Fourier infra-red spectroscopy (FTIR).

Specimens were kept in light‑proof containers prior to the testing procedure, at 37 °C for 24 h to block ambient light from initiating further post-light curing polymerization. The KBr pellet technique was used to prepare specimens for FTIR spectroscopy. Each disc layer was ground into fine powder via a mortar and a pestle over a large area to avoid heating effect that can cause an increase in the DC [14].

Cured composite powder was added to KBr with a ratio of 2%, then pressed using a hydraulic press under a load of 8 tons to produce a pellet. Since the entire 2 mm composite disc was ground into powder and used to produce the pellet, the expected DC values are thus an average across the entire disc.

Regarding uncured specimens, a specified amount of each composite type was evenly applied on thin KBr discs. DC of uncured and cured specimens (each disc layer) was assessed by Fourier transform infrared spectroscopy (FTIR) (Alpha II, Bruker, Germany) in transmittance mode from 62 scans at a wavenumber resolution of 4 cm^− 1^ and a spectrum ranging between 500 and 4000 cm^− 1^.

The DC was evaluated by measuring the difference in the absorbance intensities of aliphatic C = C (peak at 1638 cm^− 1^) and an internal standard of aromatic C = C (peak at 1608 cm^− 1^) utilizing baseline method.

The DC percentage was determined for each sample applying the following equation:

$$DC\% = [1 - \>(\>a\>/\>b\>)\>]\> \times \>100$$

Where a= (aliphatic absorption peak / aromatic absorption peak) polymer

b= (aliphatic absorption peak / aromatic absorption peak) monomer

## Flexural strength of bulk-fill composite

**Fig. 2:**
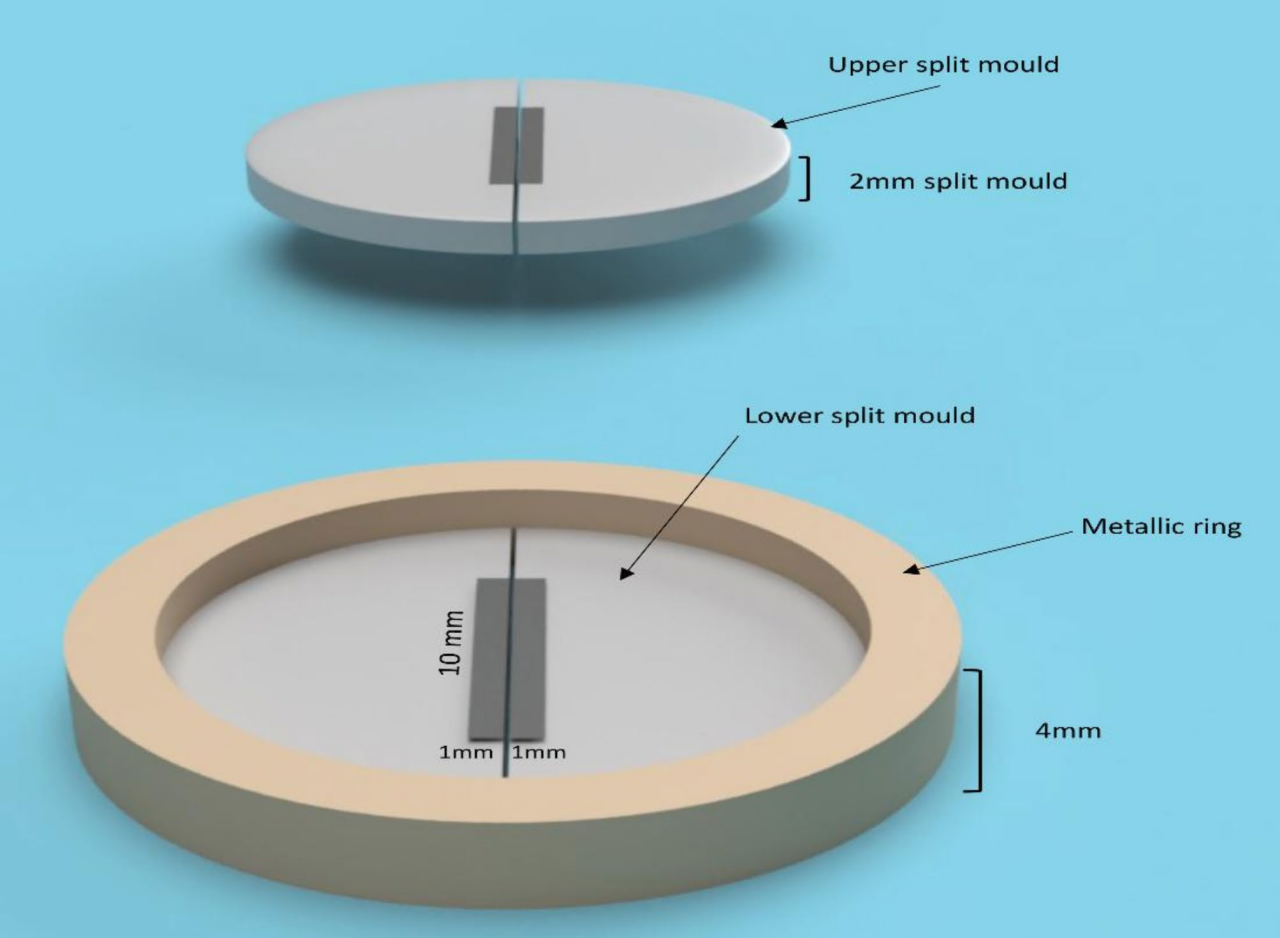
Schematic representation of molds assembly used in the construction of flexure strength specimens

Seventy-two bar-shaped specimens’ layers (2 mm X 2 mm X 10 mm) were fabricated using two split Teflon molds (2 mm thickness each) that were vertically stacked over each other as described previously in DC specimens. A metal ring of 4 mm thickness was fabricated to avoid the sliding of the molds over each other (Fig. 2). The assembly was photo-cured from the top for 20 s using non overlapping exposures to ensure homogenous polymerization throughout the specimen [15].

After light curing, two separate 2-mm thick bar-shaped specimens were obtained. This procedure was performed six times using the light curing tip with and without the presence of ICBs.

Specimens were subjected to three-point loading via a universal testing machine (Instron 3365, High Wycombe, UK) with a crosshead speed of 1 mm/min and a support span of 6 mm [15]. The flexural strength was calculated according to the equation:

$${F_S}\, = \,{{3\, \times \,F\, \times \,L} \over {2\, \times \,w\, \times \,{h^2}}}$$

where:

F_S_=Flexural strength (MPa), F = load at fracture (N), L = span between supports (mm); w = specimen length (mm); h = specimen height (mm).

### Water vapor permeability (WVP) of infection control barriers

WVP of barriers was assessed according to ASTM Standard Test Method E96 [16] also identified as the cup method. Silicone adhesive was used to properly seal the tested barriers to the mouth of a cup filled with distilled water. The cup was put inside a desiccator adjusted at 25 °C and 50% relative humidity. The water vapor transported through the film was evaluated by computing the weight alterations regularly using a sensitive 4 zero digital scale (Sartorius, Cubis^®^, Germany), until constant weight was reached for about 6 h. Weight change was plotted against time to produce a straight-line graph. The slope of the linear regression of weight loss against time was used to compute the water vapor permeability.

Water vapor permeability (WVP)(g/s·m·Pa) was calculated using the following equation: $\:WVP\:=\:CX/A\varDelta\:P$

X is the film thickness (m), A is area of the exposed film(m^2^), ΔP is the differential water vapor pressure across the film (Pa), and C is the slope of the weight loss of the dish versus time.

Thickness of ICBs used were measured using a 0–150 mm digital caliper (Durmiri, China). The test was repeated five times for each type of barrier.

### Statistical analysis

Statistical analysis was computed using SPSS (statistical package for social sciences, IBM SPSS Statistics for mac, version 24 software, Armonk, NY: IBM Corp, USA).

Data were presented as means and standard deviations. Data was checked for normality using Kolmogorov-Smirnov test and Shapiro- wilk test and were found to be normally distributed. Statistical analysis was carried out using Factorial analysis of variance (ANOVA) to explore the effect of different composite materials, infection control barriers and location of composite resin layer on flexural strength and degree of conversion. Following significant interactions, data from DC, FS and light cure irradiance were analyzed using one-way ANOVA and Tukey’s test for pairwise comparisons. Independent sample t-test was conducted to compare the effect of different bulk-fil composites and different layers of each composite on FC and DC. It was also used to compare the effect of the tested barriers on WVP.

Sample size was calculated using G*Power version 3.1.9.2 for sample size analysis at α = 0.05 and 95% power.

## Results

### Light irradiance

Light irradiance using no infection control barrier showed the highest mean value (1050 mW /cm^2^), this was followed by using sanita wrapping film (941.67 mW /cm^2^). Using pinnacle curing sleeve resulted in the lowest light irradiance mean value (850 mW /cm^2^). (Table 1)

**Fig. 3:**
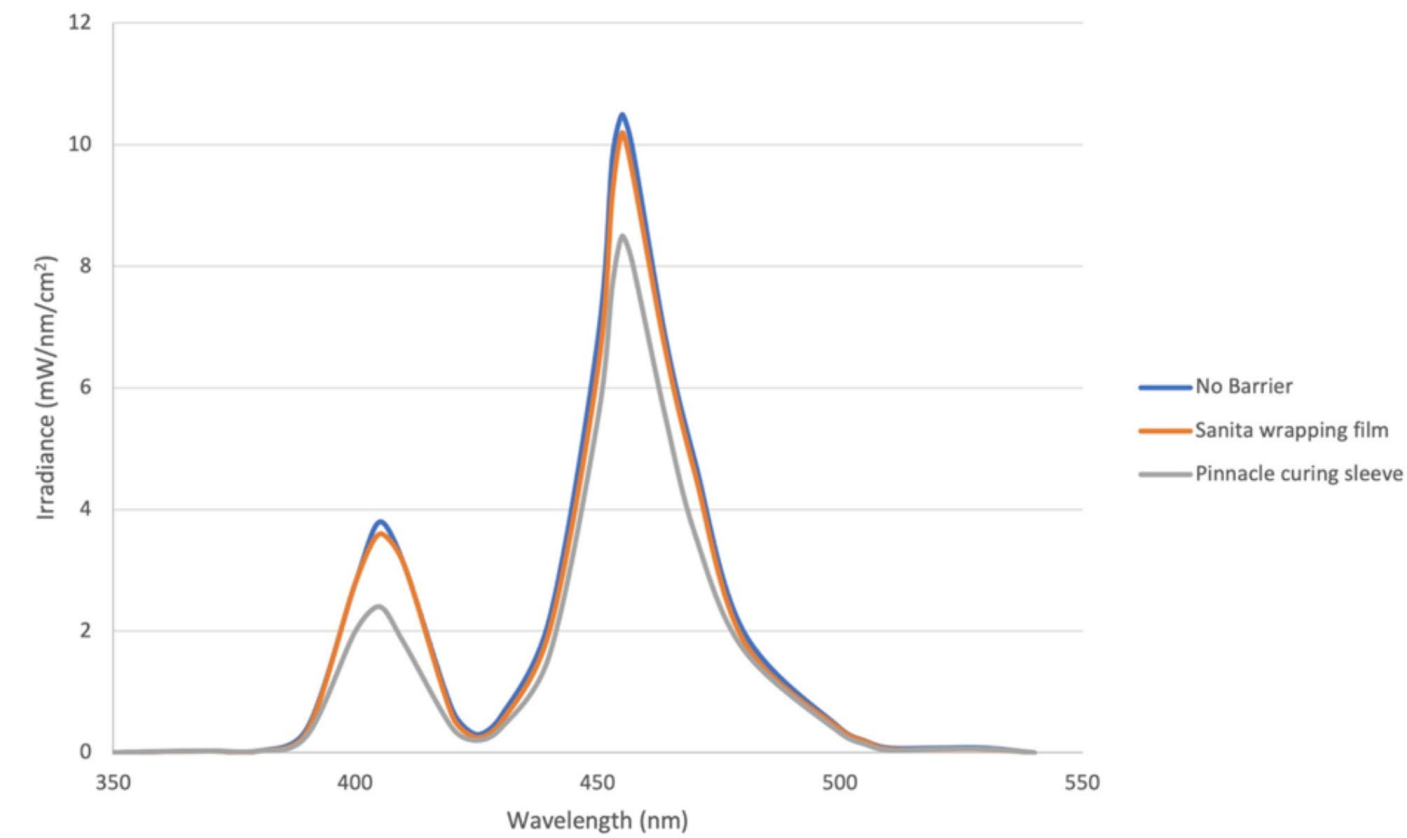
Effect of Pinnacle curing Sleeve and Sanita wrapping film on spectral irradiance from Bluephase N light cure

The spectral profile for Bluephase N light curing unit indicated the existence of emission peaks in the blue and violet regions. When using either barrier, spectral loss was detected at both regions, however Pinnacle curing sleeve resulted in a lower irradiance compared to Sanita wrapping film (Fig. 3).

**Table 1:** Mean and standard deviation values of light irradiance (mW /cm^2^) with/without infection control barriers

Light cure irradiance (mW/cm^2^)						
	Mean	Std. deviation	Std. error	95% Confidence interval for mean		P-value
				Lower bound	Upper bound	
No Barrier	1050.00^a^	31.62	12.91	1016.81	1083.19	0.0001
Pinnacle curing Sleeve	850.00^c^	31.62	12.91	816.81	883.19	
Sanita wrapping film	941.67^b^	37.64	15.37	902.17	981.17	

Different lowercase letters indicate significant difference

### Degree of conversion

Regarding the effect of the infection control barriers, Table 2 showed that light curing of either X-tra fil or Tetric N-ceram without an ICB or on using Sanita wrapping film resulted in the highest degree of conversion mean values of either the upper or lower layers of the specimens. Using Pinnacle curing sleeve produced the lowest degree of conversion mean values.

As for the location of the cured specimen layer, upper layers of composite resin showed a higher degree of conversion than lower layers for both types of composites and whether an infection control barrier was used or not (Table 2).

Concerning the effect of bulk-fill composite type, X-tra fil showed higher degree of conversion compared to Tetric N-Ceram when using an ICB or not, and for both upper and lower layers of the composite specimens (Table 2).

**Table 2:** Mean and standard deviation values of degree of conversion (%) regarding each type of composite, with / without an infection control barrier

Degree of conversion (%)				
Bulk-fill composite	Type of barrier	Layer’s location		P-value
		Upper 2 mm layer	Lower 2 mm layer	
		Mean (Standard Deviation)	Mean (Standard Deviation)	
X-tra fil	No Barrier	68.17 (0.75)^aA^	64.33 (1.51)^aB^	0.0001
	Pinnacle Sleeve	56.83 (1.83)^bA^	43.67 (1.49)^bB^	0.0001
	Sanita wrapping film	65.83 (2.79)^aA^	60.83 (3.65)^aB^	0.024
P-value		0.0001	0.0001	
Tetric N-Ceram	No Barrier	63.67 (3.20)^aA^	58.67 (3.44)^aB^	0.026
	Pinnacle Sleeve	51.00 (3.16)^bA^	39.17 (3.76)^bB^	0.0001
	Sanita wrapping film	62.00 (2.82)^aA^	56.16 (3.31)^aB^	0.008
P-value		0.0001	0.0001	

Different small letters indicate significant difference within the same column for every composite resin type. Different capital letters indicate significant difference within the same row for every barrier type

### Flexural strength

Using the LCU without an infection control barrier or while using Pinnacle curing sleeve or Sanita wrapping film resulted in flexural strength mean values of no significance difference regarding upper composite resin layer for each of X-tra fil and Tetric N-ceram. As for the composite resin lower layer, higher flexural strength mean values were observed when no barrier was used and when using the wrapping film, with no significant difference between them. Using the curing sleeve resulted in the lowest flexural strength mean value (Table 3).

Regarding the location of composite resin layers, the upper layer showed higher mean flexural strength compared to the lower layer (*p* < 0.05) for each type of bulk-fill composite and whether an infection control barrier was used or not (Table 3).

Concerning the effect of bulk-fill composite resin type, X-tra fil showed higher flexural strength mean values compared to Tetric N-Ceram (*p* < 0.01), whether an infection control barrier was used or not and for both layers of the composite resin specimen (Table 3).

**Table 3:** Flexural strength mean values (MPa) for each type of composite, with / without an infection control barrier

Flexural strength (MPa)				
Bulk-fill composite	Type of barrier	Layer’s location		P-value
		Upper 2 mm layer	Lower 2 mm layer	
		Mean (Standard Deviation)	Mean (Standard Deviation)	
X-tra fil	No Barrier	154.03 (3.04)^aA^	143.27 (5.09)^aB^	0.001
	Pinnacle sleeve	153.77 (6.01)^aA^	118.8 (3.54)^bB^	0.0001
	Sanita wrapping film	149.67 (3.61)^aA^	141.16 (4.86)^aB^	0.006
P-value		0.192	0.0001	
Tetric N-Ceram	No Barrier	134.20 (5.53)^aA^	120.13 (3.81)^aB^	0.0001
	Pinnacle sleeve	129.60 (4.57)^aA^	98.93 (8.76)^bB^	0.0001
	Sanita wrapping film	128.50 (6.77)^aA^	119.25 (5.57)^aB^	0.027
P-value		0.218	0.0001	

Different small letters indicate significant difference within the same column for every composite resin type. Different capital letters indicate significant difference within the same row for every barrier type

The significance value was set at P < 0.05.

### Water vapor permeability (WVP)

Sanita wrapping film showed higher water vapor permeability mean value (0.05 ± 0.009 g/s.m.Pa) compared to Pinnacle Cure Sleeve (0.014 ± 0.0018 g/s.m.Pa, p-value < 0.001).

## Discussion

To face the spreading of infectious diseases in dental offices, it is mandatory to select infection control barriers providing acceptable infection control measures during usage of light curing units. However, such barriers should not adversely affect the esthetic and functional performance of resin composites, especially bulk-fill types.

The concept of bulk-fill composites arose as a time saving procedure for deep cavity restoration. Depth of cure and its effect on properties were challenges facing such technique. Since light curing infection control barriers (ICBs) became obligatory for appropriate infection control, several research investigated if such barriers would influence the light intensity and hence the depth of cure. However, such studies were only limited to a 2 mm depth. Our research raises the question if such barriers would have an adverse effect at depths greater than 2 mm as in the case of bulk- fill composites.

Two types of ICBs were used in this study, a wrapping film (Sanita Consumer products), due to its availability, affordable cost, and ease of adaptability. Furthermore, such barrier can cover the whole body of the LCU, rather than just the tip, which is essential to prevent cross-infection [17]. A customized ICB ( Pinnacle Cure Sleeve) was also selected, despite being costly, it is manufactured under certain specifications to ensure proper infection control measures.

Two bulk-fill composites were selected in the present research: X-tra fil and Tetric N-Ceram. Each adopts a different strategy in overcoming the problem of insufficient degree of conversion at a 4 mm depth. One approach was to increase the fillers size accordingly, the surface area between fillers and organic matrix is reduced, thus minimizing light scattering as in X-tra fil resin composite. Another strategy to increase curing depth was embraced in tetric N-Ceram Bulk-fill by presenting an additional patented photo-initiator (Ivocerin), which is claimed to be more efficient than camphorquinone (CQ) [14].

Ivocerin absorbs light between the violet (370 nm) and blue (510 nm) ranges, with a peak absorption of 418 nm. It is more responsive than CQ since it experiences a self-cleavage process and does not require a reducing agent as the tertiary amines required for CQ [15].

Laboratory grade spectroradiometer was used to measure the irradiance of LCU as dental radiometers do not offer reliable measurements. The precision of dental radiometers differs considerably as much as 80% and is reliant on the diameter of the light guide [18].

When LCU was used with either type of ICBs, light irradiance was significantly reduced. Pinnacle Cure Sleeve caused a greater reduction of irradiance than the Sanita wrapping film. Alterations in light intensity reduction among different barriers might be due to different barriers opacities and thicknesses [19]. Pinnacle cure sleeve was found to be more opaque and thicker (0.03 mm) than a single layer of wrapping film (0.01 mm), thus causing more intensity reduction. This was in line with other studies which deduced that Pinnacle Cure Sleeve was opaquer and produced the greatest decline in the mean radiant exposure at 0◦ angulation and 0 mm distance of the LCU from the sensor. On the other hand, the minimal irradiance reduction was due to using a single layer of cling wrapping film, placed correctly without any creases or folds located on the curing tip [7, 20]. Accordingly, the first null hypothesis was rejected.

Using the Pinnacle cure sleeve resulted in the lowest degree of conversion mean values for each of X-tra fil and Tetric N-ceram in either the upper or lower 2 mm layers of the specimen. This finding was further supported by the results of light irradiance, where Pinnacle Cure Sleeve caused the largest decrease in the mean radiant exposure. Reduction in radiant exposure below 150 mW/cm^2^ was found not to compromise the curing of the composite resins [7, 8]. In the present study, Pinnacle Cure Sleeve caused a 200 mW/cm^2^ reduction in the radiant power, accordingly this resulted in a lower degree of conversion for the bulk-fill composites. As for the wrapping film, reduction in irradiance was below 150 mW/cm^2^, hence no significant decrease in DC was detected compared to using LCU without ICBs.

Furthermore, Pinnacle Cure Sleeve yielded a considerable reduction in the spectral irradiance at both the blue and violet regions compared to Sanita wrapping film. This may be another justification why Pinnacle Cure Sleeve caused a significant reduction in DC regardless of the type and layer of composite, whether the upper or lower 2 mm.

The upper 2 mm specimens’ layers of either types of composite resin showed a higher degree of conversion compared to the lower 2 mm layers with or without using ICBs. Such findings reflect the impact of composite depth on light intensity and thus degree of conversion. This was supported by Ma et al. [20], they reported that there was approximately an additional 10% drop in radiant exposure with every 2 mm increase in distance whether with or without an ICB. Yet, this study didn’t take into consideration the composite resin material which may lead to further reduction in light irradiance. Since a direct correlation exists between the microhardness of a composite and its DC, studies using Vickers microhardness to determine the curing efficiency of either X-tra-fil or Tetric N-ceram, were also in line with our results, they showed that microhardness significantly decreased with an increase in curing depth [21, 22].

As regards the type of bulk-fill composite, X-tra fil exhibited a higher degree of conversion compared to Tetric N-Ceram when using a LCU barrier or not and for both upper and lower layers of the composite specimens. A plausible explanation for such a finding might be the higher translucency of X-tra fil compared to Tetric N-Ceram Bulk-fill. This may be related to larger filler size and close matching in the refractive indices of the filler particles and resin matrix as per manufacturer’s claims, which improves the light transmission through the resin composites [23–25]. Such finding was further supported by Maghaireh et al. [26] who found that N-Ceram Bulk-fill had a lower depth of cure than that of X-tra fil, verifying that the depth of cure of CQ-based resin composites can be more than those modified by the addition of Ivocerin to CQ, accordingly the additional photoinitiator (Ivocerin) in N-Ceram Bulk-fill is not able to fully offset the lower translucency of this product. This relies on the fact that camphorquinone is activated via blue spectrum which can penetrate deeper through the composite than violet spectrum used to activate Ivocerin [27].

Another justification of the higher DC of X-tra fil is the presence of triethylene glycol dimethacrylate (TEGDMA) in its matrix composition [28]. TEGDMA is considered a diluting monomer and demonstrates the highest DC among composite resin monomers. A synergistic effect on both the DC, as well as the depth of cure can result when bis- GMA is diluted with a low viscosity monomer. Therefore, a high concentration of TEGDMA might be a reason for a high curing depth of X-tra fil composite resin [21].

Regarding flexural strength, using LCU with or without an infection control barrier produced mean values of no significant difference, regarding the upper layer of composite resin, for each of X-tra fil or Tetric N-Ceram. Such finding seems not in line with the DC results, since using Pinnacle Cure Sleeve produced lowest DC values in the upper 2 mm layer of composite resin specimens. It could be speculated that the reduction of DC while using the curing sleeve was not influential to adversely affect the flexural strength. This was in concur with a previous study that deduced a weak correlation between the DC and flexural strength [29]. Mechanical properties of resin composites do not rely only on the DC but also on other factors, most importantly filler load and resin composition [30].

As for the lower layer of the composite resin specimens, flexural strength results were in line with DC. Using Pinnacle Cure Sleeve significantly reduced the flexural strength which was the case with DC results for the lower layer of composite resin specimens. A possible explanation for such finding might be that Pinnacle Cure Sleeve reduced the DC values for both X-tra fil (43.67%) and Tetric N-Ceram (39.17%) to a level far below the achievable range of bulk-fill composites, at a depth of 4 mm and curing time 20 Sects. (50–75%) [31, 32]. Such a low percentage of DC might have adversely affected the flexural strength of composites regardless of other factors such as filler loading and matrix composition.

It is noteworthy that although using Pinnacle Cure Sleeve with either type of bulk-fill composites resulted in immediate flexural strength of acceptable values, DC values for the lower 2 mm layers were below adequate ranges. Such low DC values may lead to water sorption and disintegration of composites thus affecting their clinical performance and longevity.

The flexural strength of the upper 2 mm layer of both bulk-fill composites resin specimens was significantly higher than the lower 2 mm part whether an ICB was used or not. This might be related to multiple factors associated with the DC, one of which is the distribution pattern of porosities within the bulk-fill composite. A previous study explored the porosities distribution in bulk-fill composites and concluded that such porosities were more common in the deeper layers of composite restorations thus adversely affecting the mechanical properties at deeper layers [33].

X-tra fil showed higher flexural strength mean values compared to Tetric N-Ceram, within all testing conditions. Such finding may be related to the idea that Tetric N-Ceram produces a higher number of free radicals due to the presence of both CQ and Ivocerin as initiators. Such an increase in free radicals tends to speed the gelation time of Tetric N-Ceram compared to X-tra fil, giving less time for polymer chains to re arrange themselves forming longer polymers chains with more cross-linking [34]. Thus the resin matrix will be formed of shorter chains with less crosslinking, resulting in significantly lower mechanical properties of Tetric N-Ceram Bulk-fill compared to X-tra fil [23]. Based on the DC and flexural strength findings, the second null hypothesis was rejected.

It is noteworthy that the higher DC of X-tra fil cannot be considered a justification of its higher flexural strength compared to Tetric N-Ceram, since a reason for such higher DC was the presence of TEGDMA which is considered a flexible low molecular weight monomer which acts as a diluent. Thus, TEGDMA might adversely affect the strength of X-tra fil [32].

WVP test was chosen to assess the infection control efficiency of ICUs to reduce surface contamination of equipment developing from direct contact and aerosolized droplet particles thus combating oral bacteria and viruses’ transmission, since viral and bacterial contamination can occur via droplets and aerosols of saliva and blood [17]. This method depends on calculating the WVP of a sample by gravimetry.

Water vapor permeability (WVP) of ICBs was investigated to assess their capacity Wrapping film showed higher water vapor permeability compared to the Pinnacle curing sleeves, thus rejecting the third null hypothesis. Such findings might be affected by the difference in thicknesses in ICBs where Pinnacle was found to have a thickness of 0.03 mm while wrapping film had a thickness of 0.01 mm.

One limitation of this in vitro study was that intraoral conditions, for instance masticatory forces, moisture, along with operator variations could not be studied during evaluating the effect of ICBs on properties of bulk- fill composites. Yet this study is the first to address the effect of ICBs on bulk-fill composites, making it important to determine the effect of each variable independently, which in turn encourages performing the study primarily outside the biological context. Other limitations are that only two bulk-fill composites were tested in the study and that longevity and clinical serviceability of such bulk-fill composites were not investigated. Accordingly, further studies are required to investigate more bulk-fill composites and to determine the effect of aging on these composites when cured using ICBs on LCUs. More translucent commercially available ICBs should also be investigated in comparison with wrapping films.

## Conclusions

Within the limitations of this study the following could be concluded:

1. Using Sanita wrapping film as an infection control barrier on light curing units can be considered a cost effective, successful method to achieve infection control measures without jeopardizing the concept of bulk-fill composites.
2. Pinnacle curing sleeve can be regarded as an effective infection control barrier, however its impact on FS, DC and thus serviceability of bulk-fill composites might be questionable.

## Data Availability

Data is provided upon request from the corresponding author.

## Abbreviations

ICBs: Infection control barriers
LCUs: Light curing units
DC: Degree of conversion
FS: Flexural strength
WVP: Water vapor permeability
